# Prescribing and fitting spectacles: the role of pupillary distance and the optical centre

**Published:** 2024-05-15

**Authors:** Michelle Mehta, Stellamaris Onyegbule

**Affiliations:** 1 Lecturer in Optometry and Visual Sciences: City, University of London, London, UK.; 2 Optometrist, Skipper Eye-Q Super Speciality Eye Hospital Abuja, Nigeria.

**To ensure clear vision, it is vital that the optical centre and pupils are aligned correctly.**

The optical centre (OC) of a spectacle lens is the point at the centre of the lens through which light rays pass without any unwanted prismatic effects (displacement of the image). In other words, the optical centre of a lens is the point at which the wearer will experience the clearest vision.

It is therefore crucial for the optical centre to be correctly aligned with the wearer's pupils, so they can see clearly, no matter which direction they look in. If the optical centre is not correctly aligned, light rays that pass through the lens are refracted, or bent, which leads to blurred or distorted vision. This can cause the patient to experience eye strain, discomfort, and headaches.

The optical centre also plays an important role when prism is prescribed, usually for patients who have difficulty combining the images received from both eyes. Prisms can be created by decentring the lens in the frame; i.e., moving it so that the patient looks through the lens at a point away from the optical centre.

**Monocular or binocular pupillary distance?**

It is standard practice to measure and record both monocular and binocular PDs for each patient. Monocular PD is often recommended for greater accuracy in prescribing because most people have asymmetrical faces; i.e., our left and right pupils are not always the same distance from the centre of our nose. This asymmetry can result in wrong placement of the optical centre and induces prismatic distortion.

**Table 1:** When to measure monocular and binocular pupillary distance

	Monocular pupillary distance?	Binocular pupillary distance?
High prescription OR high facial asymmetry	Yes	No
Bifocal or progressive lenses	Yes	No
Squint	Yes	No
Low prescription + minimal facial asymmetry	Not needed	Usually sufficient
If the patient is a baby or young child	Not needed	Usually sufficient

## 1. Measuring pupillary distance

The first step in achieving correct alignment of the optical centre is to accurately measure and record the pupillary distance, measured in millimetres (mm). Depending on the circumstances and the patient (see panel) this can be either the **binocular** pupillary distance (the distance between the centres of the two pupils), or the **monocular** pupillary distance: the distance between the centre of the pupil and the centre of the nose, recorded for each eye separately (see panel).

These are the measurements the laboratory will use when fitting lenses to the patient's chosen frame. If the pupillary distance is measured incorrectly, the optical centre will be incorrectly set within the spectacle frames, which can only be rectified by remeasuring the pupillary distance correctly, and remaking the spectacles.

**NOTE:** It is important to guide patients in their choice of frame. If the frame is too big or too small, or if it has been poorly adjusted, this can lead to misalignment of the optical centre, even when the pupillary distance has been measured correctly.

### What you will need:

- A pupillary distance ruler

### Before you begin:

- Wash your hands and tell the patient what you are going to do.
- Position yourself directly in front of the patient, ensuring that you are at the same height as them, and about an arm's length in distance.

### Measuring the binocular pupillary distance

1. Holding the ruler between your thumb and forefinger, place the ruler so that it is resting on the bridge of the patient's nose and forehead. Use your middle finger to steady your hand on their head.
2. Close your right eye and ask the patient to look towards your open left eye.
3. Place the zero reading of the ruler so that it is aligned with the centre of the patient's right pupil.
4. Keeping the ruler in this position, close your left eye and ask the patient to now look towards your open right eye.
5. Read the measurement that aligns with the pupil centre of their left eye. This is the binocular pupillary distance.
6. If the patient has very dark irises, making it difficult to see the pupil, you can measure from the temporal edge of the right iris to the nasal edge of the left iris, using the same technique.
7. Record this distance in the notes.

### B. Measuring the monocular pupillary distance in each eye

1. Measure from the centre of the right pupil to the centre of the patient's nose and record the **right monocular pupillary distance** in the prescription notes.
2. Measure from the centre of the nose to the centre of the left pupil and record the **left monocular pupillary distance** in the prescription notes.

## 2. Marking the pupillary distance on the dummy lenses

In situations where a progressive lens is prescribed, in particular, it is important to measure and mark the position of the pupil on the dummy lenses in the frame the patient has chosen. This will ensure that the wearer is looking through the right part of the lenses for distance, intermediate, and near vision.

**Figure 1:**
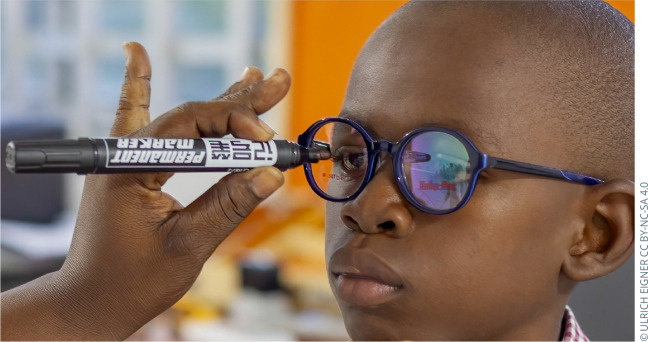
Marking the position of the pupil on dummy lenses. mozambique

1. The patient should wear the frame chosen at the position they would be comfortable wearing their spectacles.
2. Sit in front of the patient and ask them to look straight ahead.
3. Position yourself with your eyes level with the patient's.
4. When marking the right lens, tell the patient to look towards your open left eye, and vice versa.
5. Keeping you head still, make a dot on the dummy lens directly in line with the centre of the pupil (Figure 1).

## 3. Finding the optical centre

Once spectacles come back from the laboratory, and before fitting the frame on the patient, the next step is to find the optical centre. There are two ways of doing this:

### a. Locating the optical centre using a focimeter.

You can use a focimeter to locate the optical centre of a lens if you have access to one, using the following steps.

**Figure 2:**
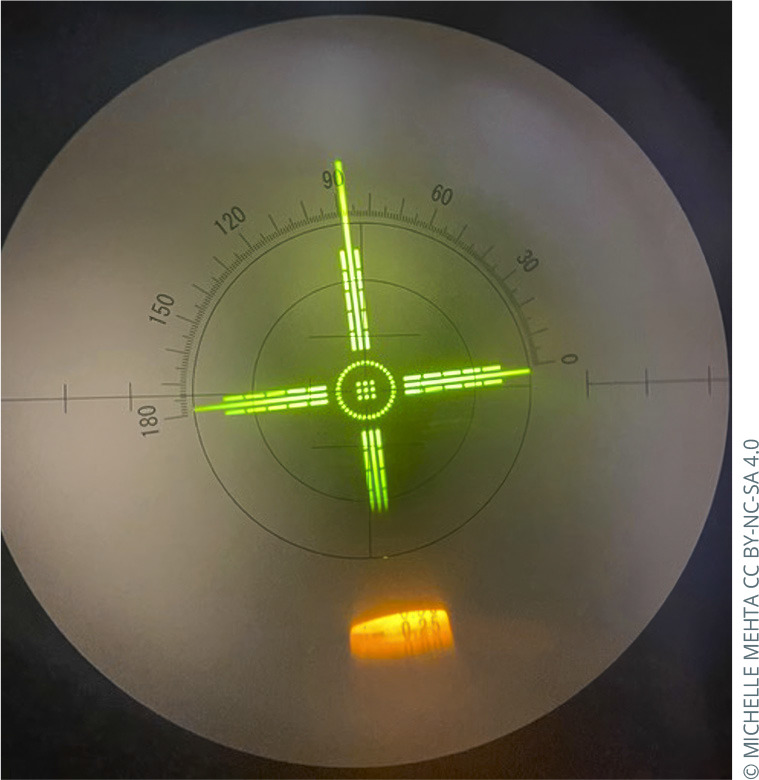
The optical centre has been located using a focimeter

1. First focus the machine. Turn the eyepiece fully anticlockwise. Then, while looking into the focimeter, turn the eyepiece clockwise until the graticule seen inside is just clear. At this point, the focimeter is focused for your eye.
2. Check the calibration of the focimeter by turning the power wheel on the side until the target is completely clear. If, at this point, the reading of the power is at 0.00D, then your machine is correctly calibrated. If not, then this would need to be taken into account when using the focimeter to measure a spectacle prescription.
3. Place the spectacles in the focimeter so that they are facing away from you and completely flat on the frame table.
4. To locate the optical centre, move the spectacles up, down and side to side, while looking through the eyepiece, until the target (in green) is in the centre of the black graticule (Figure 2).
5. Now you can clamp the spectacles in place using the spectacle clamp.
6. Mark the optical centre using the marking device found on the right-hand side of the focimeter.

It is usually good practice to begin with the right lens first, and then move on to the left lens, in order to maintain consistency.

### b. Locating the optical centre using hand neutralisation

**Figure 3:**
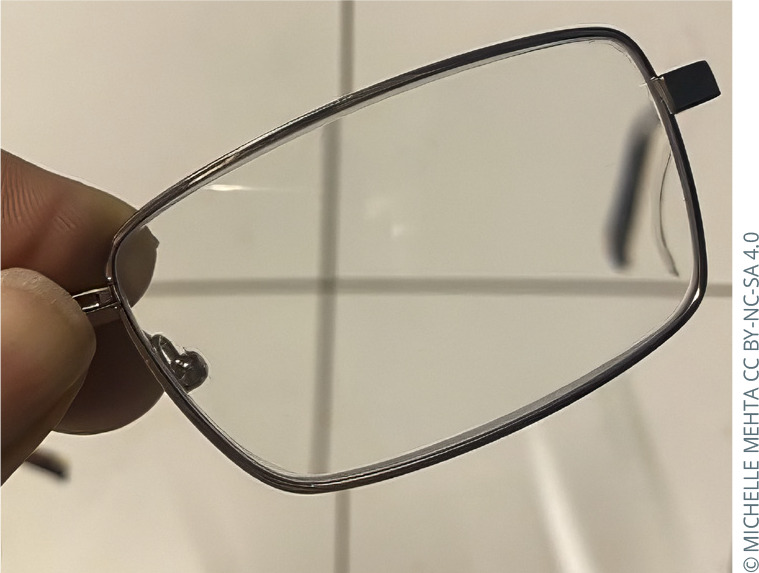
Lens not quite aligned with a cross chart

**Figure 4:**
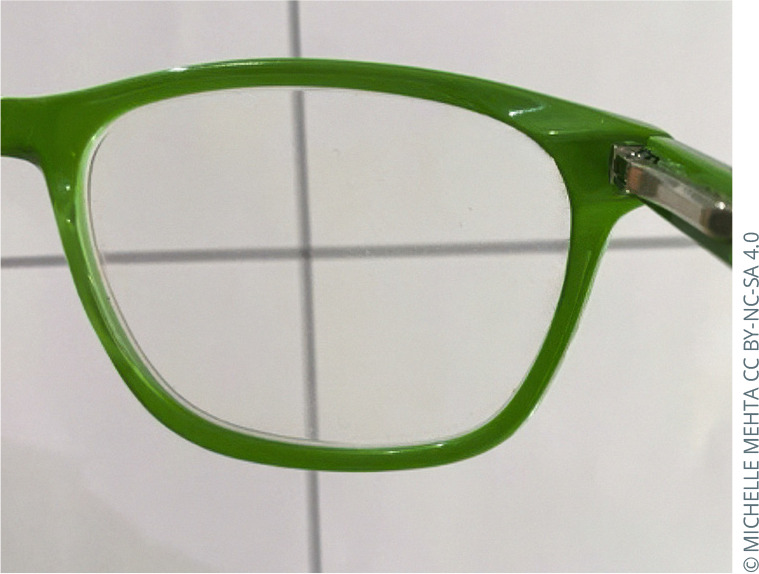
Lens aligned with a cross chart

If you do not have access to a focimeter, you can use hand neutralisation to locate the optical centre of a spectacle lens, using the following steps:

1. Draw a cross on a piece of paper, with the lines at 90 degrees to each other, and stand it up against a wall.
2. Look at the cross through the lens on which you are locating the optical centre, while holding it about arm's length away (Figure 3).
3. Move the lens up, down, left, and right, and turn it clockwise and anticlockwise – until the lines that you can see through the lens are exactly in line with the lines that remain outside the lens (Figure 4).
4. When you have reached this point, the centre of the cross will indicate the optical centre. You can then mark this using a fine marker pen.

## 4. Fitting spectacles

The final step is to adjust the spectacles to ensure a good fit and check that the optical centre – now marked on the spectacles – matches the patient's pupils.

Regular adjustment of spectacle frames, to ensure a good fit, will ensure that the optical centres are always correctly aligned with the patient's pupils.

